# Genetic and pharmacological inhibition of calcineurin corrects the BDNF transport defect in Huntington's disease

**DOI:** 10.1186/1756-6606-2-33

**Published:** 2009-10-27

**Authors:** Jose R Pineda, Raúl Pardo, Diana Zala, Hua Yu, Sandrine Humbert, Frédéric Saudou

**Affiliations:** 1 Institut Curie, Unité Mixte de Recherche 146, F-91405 Orsay, France; 2 Centre National de la Recherche Scientifique, Unité Mixte de Recherche 146, F-91405 Orsay, France

## Abstract

**Background:**

Huntington's disease (HD) is an inherited neurogenerative disease caused by an abnormal expansion of glutamine repeats in the huntingtin protein. There is currently no treatment to prevent the neurodegeneration caused by this devastating disorder. Huntingtin has been shown to be a positive regulator of vesicular transport, particularly for neurotrophins such as brain-derived neurotrophic factor (BDNF). This function is lost in patients with HD, resulting in a decrease in neurotrophic support and subsequent neuronal death. One promising line of treatment is therefore the restoration of huntingtin function in BDNF transport.

**Results:**

The phosphorylation of huntingtin at serine 421 (S421) restores its function in axonal transport. We therefore investigated whether inhibition of calcineurin, the *bona fide *huntingtin S421 phosphatase, restored the transport defects observed in HD. We found that pharmacological inhibition of calcineurin by FK506 led to sustained phosphorylation of mutant huntingtin at S421. FK506 restored BDNF transport in two complementary models: rat primary neuronal cultures expressing mutant huntingtin and mouse cortical neurons from *Hdh*^Q111/Q111 ^HD knock-in mice. This effect was the result of specific calcineurin inhibition, as calcineurin silencing restored both anterograde and retrograde transport in neurons from *Hdh*^Q111/Q111 ^mice. We also observed a specific increase in calcineurin activity in the brain of *Hdh*^Q111/Q111 ^mice potentially accounting for the selective loss of huntingtin phosphorylation and contributing to neuronal cell death in HD.

**Conclusion:**

Our results validate calcineurin as a target for the treatment of HD and provide the first demonstration of the restoration of huntingtin function by an FDA-approved compound.

## Background

An abnormal polyglutamine (polyQ) expansion in the N-terminal part of the huntingtin protein causes Huntington's disease (HD), a fatal neurodegenerative disorder characterized by the dysfunction and death of striatal and cortical neurons in the brain [1]. HD is characterized by motor, cognitive and psychiatric symptoms and the age at onset is inversely correlated with the number of CAGs encoding glutamines in the huntingtin protein. There is currently no effective treatment for preventing the death of neurons in the brain or disease progression. Promising treatment strategies involve the identification of compounds capable of restoring functions altered in disease [2].

The mechanisms underlying neuronal dysfunction and death in HD are complex and involve both a gain of new toxic functions and a loss of the neuroprotective functions of wild-type huntingtin [1]. Several groups have demonstrated changes in the microtubule (MT)-dependent transport of vesicles, such as those containing brain-derived neurotrophic factor (BDNF), in diseased neurons [3-7]. This trafficking defect is an early pathogenic event and is linked to the association of huntingtin with components of the molecular motor machinery [3,8-13] and its function as a direct regulator of MT-dependent transport in different cell type including neurons [3,10,12].

Huntingtin phosphorylation at S421 abolishes the toxicity of mutant huntingtin *in vitro *and *in vivo *[14,15]. We recently demonstrated that the phosphorylation of mutant huntingtin at the S421 residue promotes neuroprotection in HD, by restoring huntingtin function in the transport of BDNF [16]. In particular, we found that pathogenic polyQ-huntingtin with an S421 mutation mimicking constitutive phosphorylation transports vesicles as efficiently as the wild-type protein. However, the potential benefits of drugs promoting huntingtin S421 phosphorylation and abolishing the transport defect in HD remain to be evaluated. Huntingtin phosphorylation at S421 is induced by the IGF-1/Akt pathway and inhibited by calcineurin [14,15]. Lower than normal levels of huntingtin phosphorylation are found in various HD models [15,17]. These lower levels of phosphorylation may be due to changes in Akt during disease progression, as observed in animal models and in the brains of HD patients [14,18] and/or an increase in calcineurin activity [15]. Consistent with this hypothesis, calcineurin levels have been found to be higher than normal in neuronal cells immortalized from HD mice [19]. A decrease in the levels of RCAN1-1L, a negative regulator of calcineurin, in the brains of HD patients may also account for the lower levels of huntingtin phosphorylation observed [20]. These observations suggest that calcineurin inhibition may be of benefit in the treatment of HD.

Calcineurin is a serine-threonine phosphatase that is highly abundant in neuronal tissues. It consists of a calmodulin-binding 60 kDa catalytic subunit, calcineurin A (CaNA), and an intrinsic Ca^+2^-binding 19 kDa regulatory subunit, calcineurin B (CaNB) [21-24]. The C-terminal part of CaNA contains autoinhibitory and calmodulin-binding domains and this subunit is regulated by various endogenous regulators, including RCAN proteins [25-30]. Calcineurin is also efficiently blocked by FK506, an immunosuppressive drug that must bind to FK506-binding proteins to exert its effects. FK506 has been shown to be neuroprotective in various neurodegenerative paradigms [31,32].

In this study, we investigated the potential value of calcineurin as a target for the treatment of HD by pharmacological and silencing approaches. Calcineurin activity was found to be dysregulated in the brains of HD mice. FK506 and siRNAs targeting calcineurin increased huntingtin phosphorylation, restoring the capacity of this protein to transport BDNF in neurons to levels similar to those in the wild-type. Thus, drugs or pathways blocking calcineurin activity are of potential interest for the treatment of HD.

## Results

### FK506 increases huntingtin phosphorylation at S421 in primary cortical neurons from *Hdh*^Q111/Q111 ^mice

**Figure 1:**
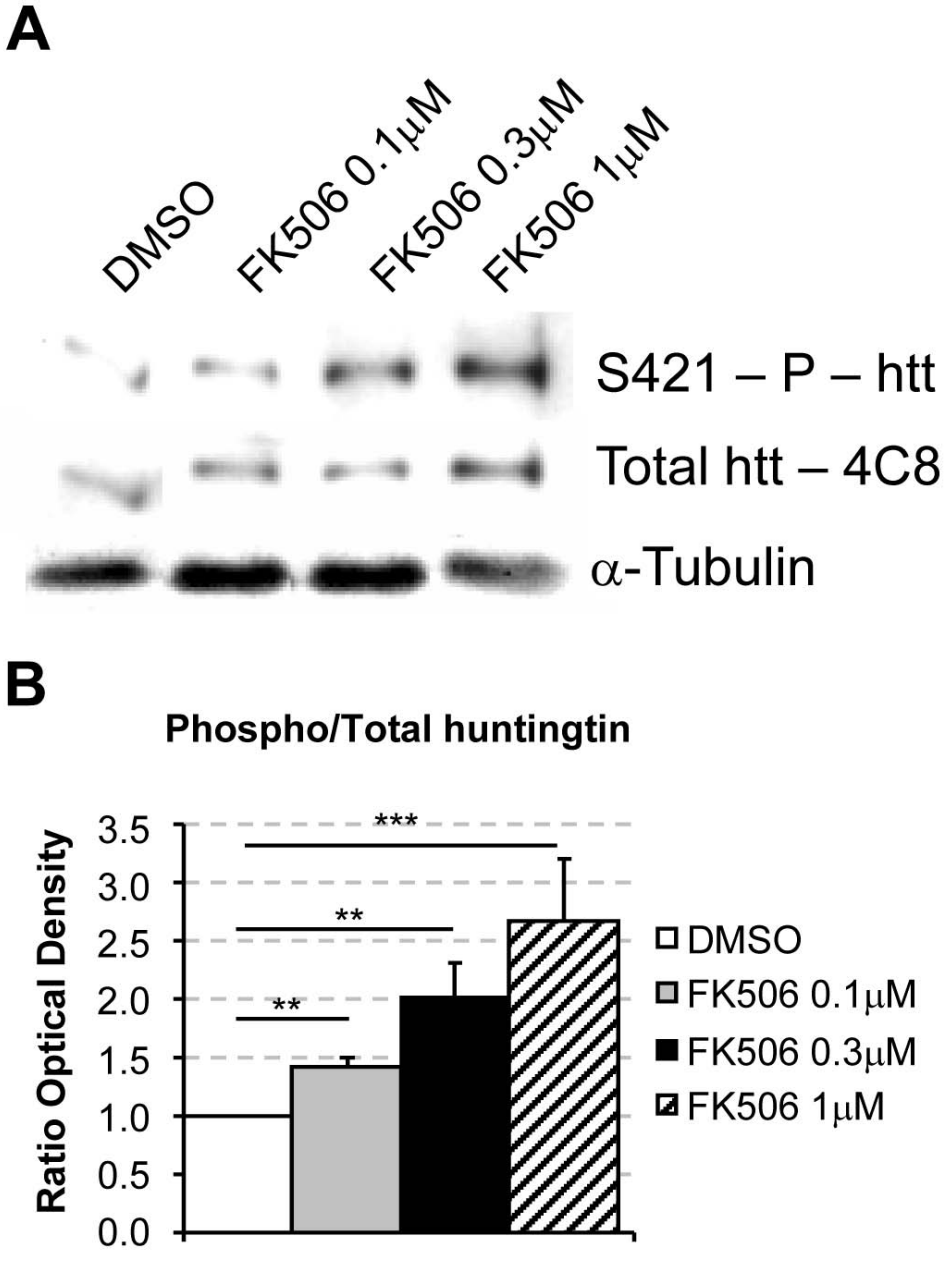
**FK506 increases huntingtin phosphorylation at S421 in primary cultures of mouse *Hdh*^Q111/Q111 ^cortical neurons**. A) 3 DIV cortical primary neurons were treated with DMSO or different FK506 concentrations (0.1 μM, 0.3 μM and 1 μM) for 1 hour and processed for Western blotting analysis with anti-phospho-huntingtin-S421-714, anti-huntingtin mAb4C8 or anti-α-tubulin antibodies. (B) Graph represents the quantitative assessment of the optical density of phosphorylated huntingtin on three independent experiments and is expressed as the phospho-huntingtin/total huntingtin ratio normalised to total huntingtin. O.D. ratios, DMSO versus FK506 0.1 μM and 0.3 μM (**p < 0.01), FK506 1 μM (**p < 0.001).

Previous studies have demonstrated that calcineurin dephosphorylates the S421 residue of huntingtin in rat cultures *in vitro *and that calcineurin inhibition results in an increase in huntingtin phosphorylation in transfected cells [15]. As a first step towards validating calcineurin as a treatment target, we investigated whether endogenous mutant huntingtin could be phosphorylated in neurons. We established primary cultures of neurons from *Hdh*^Q111/Q111 ^embryos and analyzed the extent to which various concentrations of FK506 induced the selective phosphorylation of huntingtin at S421, using a highly specific S421-phospho-htt antibody [15]. The treatment of primary neurons for one hour with FK506 at a concentration of at least 0.3 μM induced a significant increase in the selective phosphorylation of endogenous mutant huntingtin, as shown by Western blotting (Figure 1A). We expressed huntingtin phosphorylation as the ratio of phosphorylated to total huntingtin, using α-tubulin as a loading control, and found that increasing FK506 concentration induced progressive, strong phosphorylation at S421. A significant increase in phosphorylation was observed from concentrations as low as 0.1 μM (Figure 1B).

### FK506 corrects the polyQ-huntingtin-induced defect in BDNF transport in rat cortical neurons

**Figure 2:**
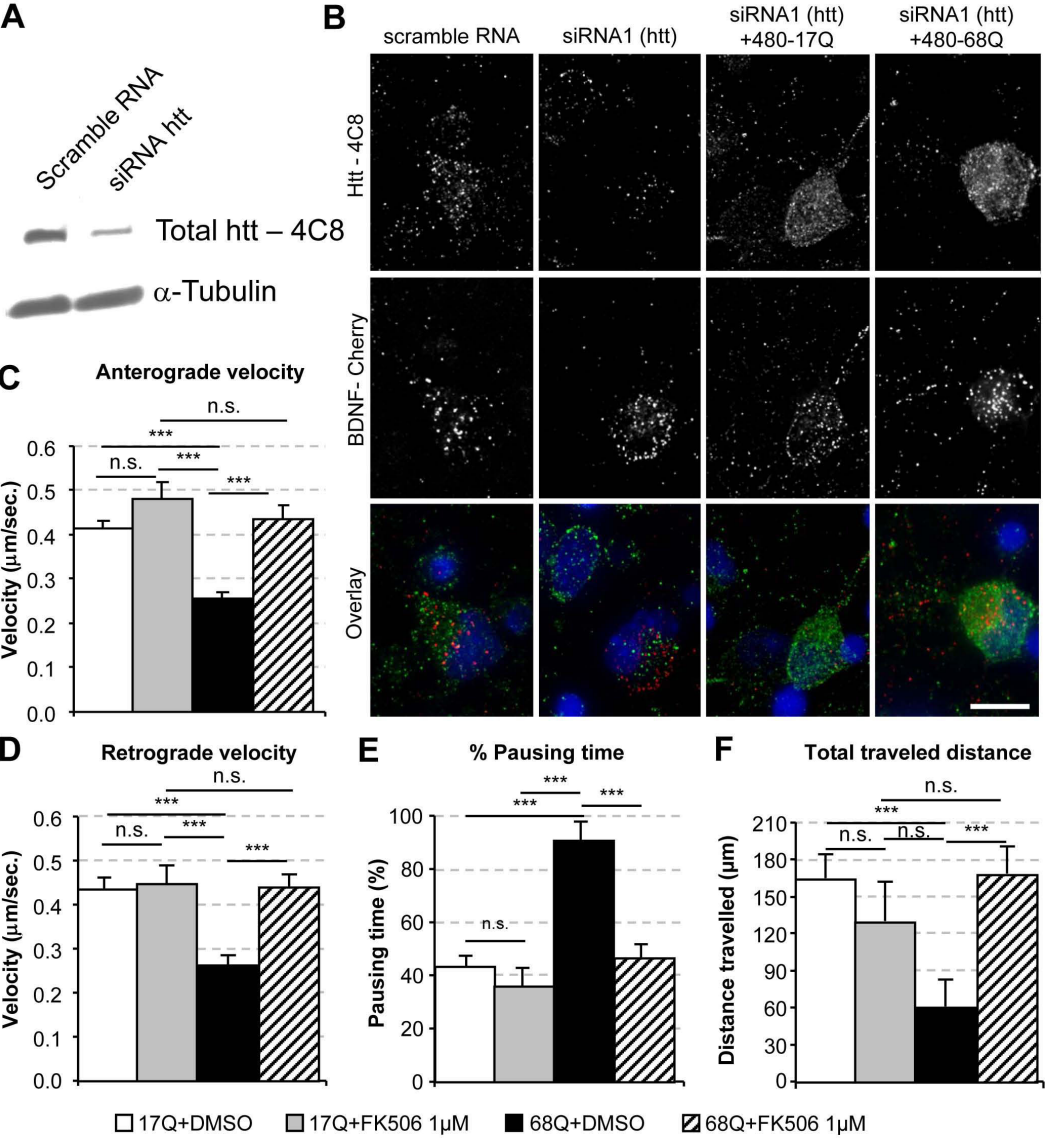
**FK506 restores polyQ-induced alteration of BDNF-vesicular transport in rat cortical neurons**. A) Rat cortical neurons were electropored to silence endogenous huntingtin and samples were analyzed by Western blotting for huntingtin expression. (B) Immunostaining of electroporated cortical neurons silenced or not for endogenous huntingtin and ectopically expressing the first 480 amino acids of huntingtin containing 17Q repeats (480-17Q, normal) or 68Q repeats (480-68Q, mutant). Whereas huntingtin silencing depletes efficiently endogenous huntingtin in individual neurons, it does not impair the re-expression of the various huntingtin constructs nor the expression of BDNF-mCherry. (C and D) FK506 treatment at 1 μM concentration restores both anterograde and retrograde transport of BDNF vesicles to wild type values (anterograde ***p < 0.0001 and retrograde ***p < 0.0001) but has no significant effect on the wild-type huntingtin fragment (anterograde velocity p = 0.10; NS and retrograde velocity p = 0.78; NS). (E) Pausing time altered in 480-68Q condition is reduced by FK506 treatment (***p < 0.0001) without having any effect in wild-type condition (p = 0.38; NS). (F) Total traveled distance of BDNF-Cherry tagged vesicles are significantly decreased in 480-68Q expressing neurons (***p < 0.001) and restored by FK506 treatment (***p < 0.001). Data are from three independent experiments: 6850 tracks, 24 neurons for 480-17Q with DMSO, 1383 tracks, 8 neurons for 480-17Q with FK506 1 μM, 3934 tracks, 15 neurons for 480-68Q with DMSO, 4910 tracks, 19 cells for 480-68Q with FK506 1 μM. Scale bar corresponds to 10 μm.

We previously demonstrated that a mutation mimicking constitutive phosphorylation at S421 (a serine to aspartic acid substitution) restored the capacity of transfected mutant polyQ-huntingtin to transport BDNF at velocities similar to those achieved by the wild-type protein [13]. We investigated whether FK506 could rescue the transport defect induced by the polyQ expansion, by analyzing the dynamics of BDNF-mCherry-containing vesicles in rat primary cultures by fast 3D videomicroscopy followed by deconvolution, as previously described [3,12,13,33]. Video experiments were performed three days after the electroporation of primary cultures of embryonic E17 rat cortical neurons. We checked that the differences in transport observed were due to the effect of the drug on the N-ter fragment of huntingtin generated from the construct used for transfection (either wild-type or mutant polyQ) rather than the endogenous wild-type huntingtin present in rat cortical neurons, by first decreasing endogenous huntingtin levels by RNA interference with an siRNA targeting the mouse huntingtin sequence (siRNA1) (Figure 2A). This "replacement" strategy has previously been used to demonstrate that siRNAs targeting huntingtin have no off-target effects and that the wild-type huntingtin N-terminal fragment reproduces the transport function of the full-length protein [13]. We found that the siRNA targeted the endogenous mouse huntingtin specifically, with no effect on the expression of the various transgenes (Figure 2B).

We then investigated the effects of the various constructs on BDNF vesicular transport. As previously reported [3,12,13,33], an N-terminal 480-amino acid fragment of huntingtin with a wild-type glutamine stretch (480-17Q) stimulated transport, whereas this function was lost if the huntingtin contained the pathological expanded polyQ stretch (Figure 2C-F). In particular, we observed significant effects of the polyQ expansion on both antero- and retrograde movement and on other dynamic parameters, such as the percentage of pausing time (Figure 2E) and the total distance traveled (Figure 2F). Our findings confirm that the wild-type huntingtin fragment reproduces the transport function of the full-length protein [13]. We next investigated whether FK506 could correct the mutant huntingtin-induced transport defect. Neurons were maintained in Neurobasal B27 serum-free medium to prevent high basal levels of huntingtin phosphorylation at S421. One hour of treatment with FK506 at a concentration inducing maximal levels of huntingtin phosphorylation (Figure 1A) restored dynamic parameters to control levels (Figure 2C-F and Supplemental Movie 1). In particular, FK506 significantly increased BDNF transport velocities in 480-68Q expressing neurons to 480-17Q levels, for both anterograde values (480-17Q + DMSO: 0.41 ± 0.02 μm/s; 480-68Q + DMSO: 0.26 ± 0.02 μm/s; 480-68Q + FK506 1 μM: 0.45 ± 0.03 μm/s; Tukey HSD p < 0.0002)(Figure 2C) and retrograde values (480-17Q + DMSO: 0.45 ± 0.07 μm/s; 480-68Q + DMSO: 0.27 ± 0.02 μm/s; 480-68Q + FK506 1 μM: 0.45 ± 0.07 μm/s; Tukey HSD p < 0.0002) (Figure 2D). FK506 treatment also significantly decreased the pausing time of 480-68Q-electroporated neurons (91.55 ± 6.87%) to a value (47.46 ± 4.84%) within the range for 480-17Q electroporated neurons (44.18 ± 3.80%, Tukey HSD p < 0.0002) (Figure 2E). Strikingly, we also observed a significant effect of FK506 on the total distance covered by vesicles (Figure 2F).

The addition of 1 μM FK506 to cells expressing wild-type huntingtin did not significantly increase anterograde or retrograde velocities, consistent with huntingtin being highly phosphorylated in wild-type conditions, with no further phosphorylation possible upon calcineurin inhibition (Figure 2C-F). Thus, FK506 corrects the BDNF transport defect in neurons expressing mutant huntingtin.

### FK506 restores BDNF transport in cortical neurons from *Hdh*^Q111/Q111 ^mice

**Figure 3:**
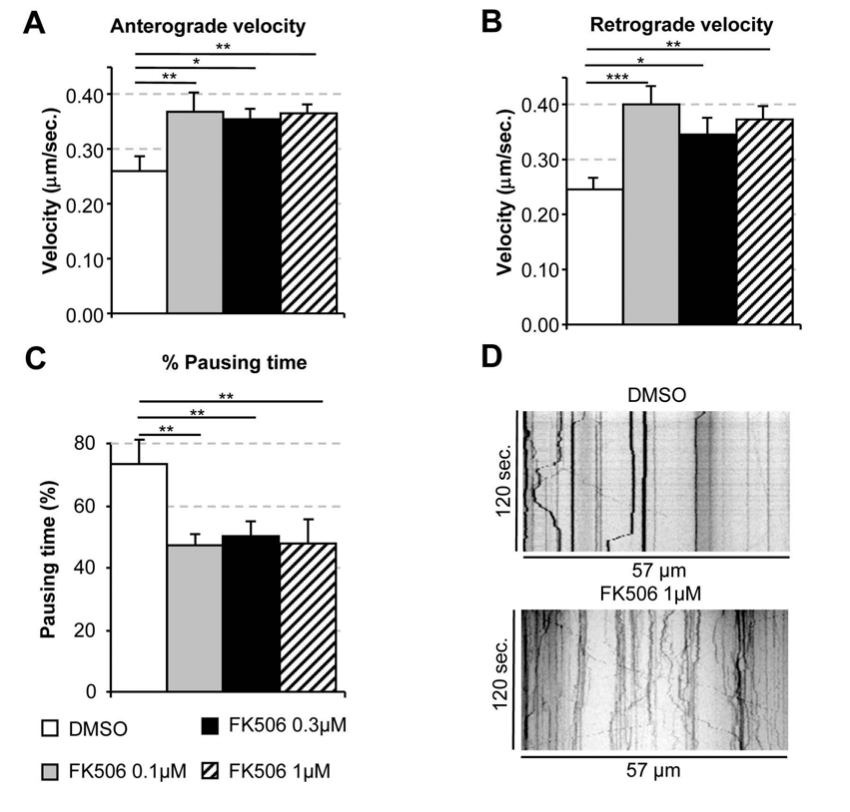
**FK506 restores transport alteration of BDNF-containing vesicles in cortical *Hdh*^Q111/Q111 ^mice neurons**. (A and B) Cortical primary neurons from knock-in Huntington's disease mice model were electropored with BDNF-mCherry and incubated for 72 h. 1 h prior videomicroscopy experiment, neurons were treated with either DMSO or the following increasing concentrations of FK506 0.1 μM, 0.3 μM 1 μM or DMSO. FK506 induced a statistically significant increase in both anterograde and retrograde velocities with all the tested concentrations (Anterograde: **p < 0.01 for 0.1 μM, *p < 0.05 for 0.3 μM **p < 0.01 for 1 μM. Retrograde: ***p < 0.001 for 0.1 μM, *p < 0.05 for 0.3 μM **p < 0.01 for 1 μM). No significant differences were observed between the different concentrations. (C) FK506 treatment also reduced significantly the pausing time of BDNF-mCherry vesicles observed in *Hdh*^Q111/Q111 ^mice cortical neurons (**p < 0.01 for 0.1 μM, 0.3 μM and 1 μM FK506 concentrations). Data are from two independent experiments, 3736 tracks, 8 cells for *Hdh*^Q111/Q111 ^+ DMSO, 4302 tracks, 10 cells for *Hdh*^Q111/Q111 ^+ FK506 0.1 μM, 2959 tracks, 11 cells for *Hdh*^Q111/Q111 ^+ FK506 0.3 μM, 2964 tracks, 8 cells for *Hdh*^Q111/Q111 ^+ FK506 1 μM. (D) Representative kymographs of BDNF-mCherry dynamics in cortical neurons from *Hdh*^Q111/Q111^mice treated with DMSO (upper kymograph) or 1 μM FK506 during 30 min (lower kymograph).

We tried to extend our findings to more physiological conditions and to determine the minimal concentration of FK506 required to rescue axonal transport, by culturing cortical primary neurons from *Hdh*^Q111/Q111 ^mice and treating them with various concentrations of FK506. FK506 concentrations as low as 0.1 μM were sufficient to increase both anterograde and retrograde transport significantly (Figure 3A-B). Indeed, DMSO-treated neurons displayed transport velocities of about 0.25 ± 0.02 μm/s, whereas neurons treated with 0.1 μM FK506 displayed faster anterograde transport, at a velocity of 0.37 ± 0.04 μm/s (p < 0.006), and retrograde transport, at a velocity of 0.40 ± 0.04 μm/s (p < 0.0005). FK506 also significantly reduced the pausing time (Figure 3C). FK506 treatment significantly increased the dynamics of BDNF-containing vesicles in *Hdh*^Q111/Q111 ^neurons as shown on representative movies (Supplemental Movie 2) and the corresponding kymographs (Figure 3D). Interestingly, the values obtained after treatment were similar to those for neurons from wild-type animals (Supplemental Figure 1). Furthermore, we observed no significant difference in transport velocity between the three concentrations (Figure 3A-B), suggesting levels of huntingtin phosphorylation sufficiently high to restore transport were achieved at a concentration of 0.1 μM. Finally, as previously reported for electroporated rat neurons (Figure 2), FK506 had no effect in wild-type conditions (Supplemental Figure 1). Thus, pharmacological treatment with FK506 corrects the transport defect in HD cortical neurons.

### FK506 inhibits calcineurin activity in cortical neurons from *Hdh*^Q111/Q111 ^mice

**Figure 4:**
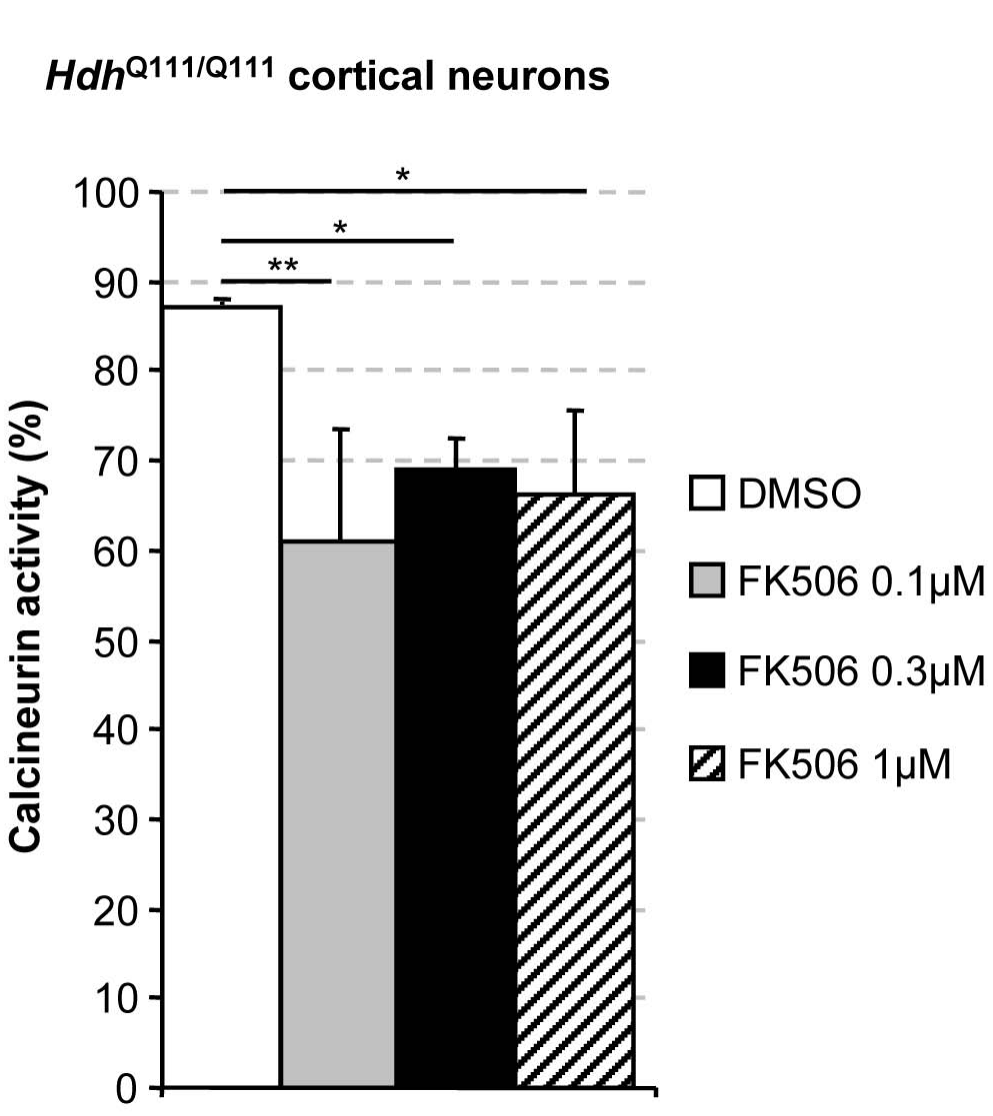
**FK506 potently inhibits calcineurin activity in *Hdh*^Q111/Q111 ^neurons**. Cortical primary cultures of neurons from knock-in Huntington's disease mice were plated with B27 media for 72 h and next treated with various concentrations of FK506, 1 μM, 0.3 μM or 0.1 μM. After 1 h of incubation cells were lysed and processed to determine calcineurin activity. Results show a significant reduction in calcineurin activity in HD neurons for all tested concentration with no significant differences between concentrations. Results were expressed as percentage of calcineurin activity per gram of tissue. (**p < 0.01 for 1 μM, *p < 0.05 for 0.3 μM and 0.1 μM FK506 concentrations).

We then investigated whether FK506 treatment, which induced a significant increase in huntingtin phosphorylation and corrected the axonal transport defect due to mutant huntingtin, led to calcineurin inhibition. We cultured cortical primary neurons from *Hdh*^Q111/Q111 ^mice and treated them with various concentrations of FK506 (0.1-1 μM). After 1 h of incubation with FK506 or DMSO, the cells were lysed, ultracentrifuged and processed for the detection of calcineurin activity (Figure 4). Calcineurin was significantly inhibited at concentrations as low as 0.1 μM, a concentration inducing significant levels of huntingtin S421 phosphorylation and the recovery of axonal transport. Together with our previous results, this indicates that pharmacological inhibition of calcineurin by FK506 induces polyQ-huntingtin phosphorylation and the restoration of vesicular transport.

### Genetic inhibition of calcineurin restores BDNF transport in cortical neurons from *Hdh*^Q111/Q111 ^mice

**Figure 5:**
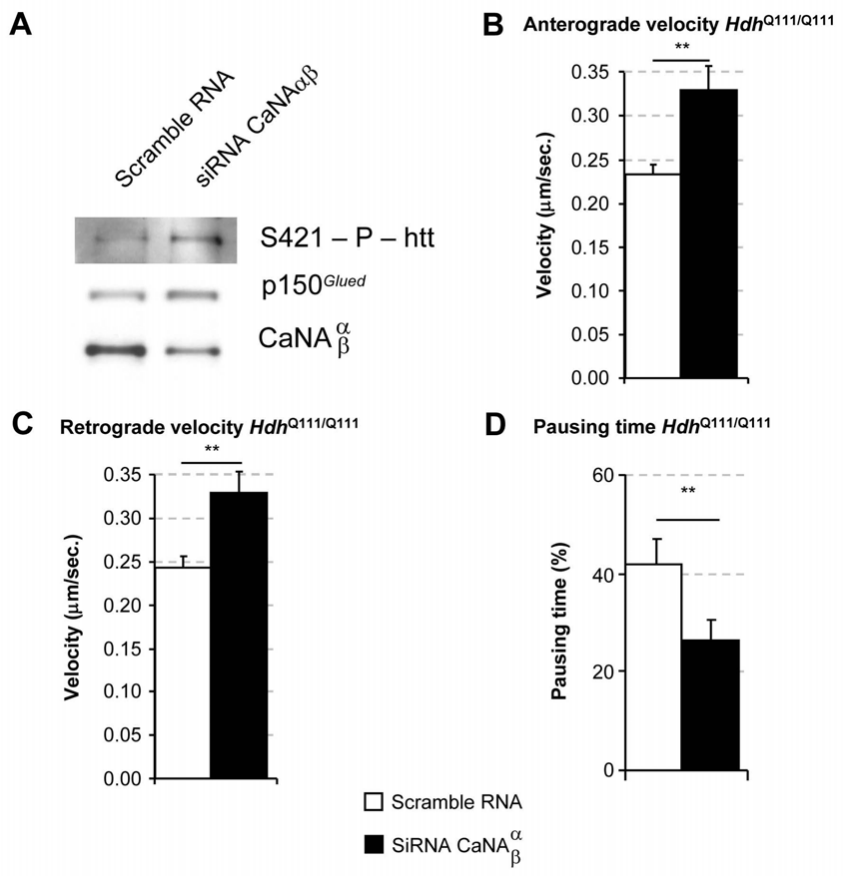
**Silencing of calcineurin by RNA interference in cortical neurons from *Hdh*^Q111/Q111 ^mutant mice restores BDNF transport**. A) Cortical primary HD neurons were electroporated with BDNF-mCherry plasmid and siRNAs against the isoforms α and β of the calcineurin A subunit. 48 h after electroporation, half of the neurons were lysed and extracts were analyzed by western blot for siRNA efficiency and huntingtin phosphorylation and, the other half of neurons were analyzed by videomicroscopy (B-D). (B and C) Calcineurin depletion increase significantly anterograde (**p < 0.01) and retrograde (**p < 0.01) velocity of BDNF vesicles. (D) Pausing time was reduced (**p < 0.01) in this mutant mice. Data are from two independent experiments, 5550 tracks, 20 cells for scramble RNA and 3461 tracks, 16 cells for siRNA calcineurin in *Hdh*^Q111/Q111 ^neurons.

For the unequivocal demonstration that calcineurin is a *bona fide *target for restoring BDNF transport, we tried to inactivate calcineurin selectively by RNA interference approaches. We electroporated primary cultures of cortical neurons from *Hdh*^Q111/Q111 ^mice with an siRNA targeting the α and β isoforms of calcineurin. We previously demonstrated that the targeting of both isoforms was required to achieve significant silencing of calcineurin in cortical neurons from wild-type mice [15]. Calcineurin silencing in neurons from *Hdh*^Q111/Q111 ^mice was maximal 48 h after electroporation. This time peak in calcineurin silencing coincided with a significant increase in huntingtin phosphorylation at S421 (Figure 5A). We used p150^*Glued*^, a subunit of dynactin, as a loading control. We then analyzed the dynamic parameters of BDNF vesicle transport in *Hdh*^Q111/Q111 ^cortical neurons depleted of calcineurin. We observed a significant increase in BDNF dynamics, as shown by the increase in anterograde transport (compare scramble RNA: 0.23 ± 0.01 μm/s with calcineurin αβ siRNA: 0.33 ± 0.03 μm/s) (Figure 5B) and in retrograde transport (compare scramble RNA: 0.23 ± 0.01 μm/s with calcineurin αβ siRNA: 0.33 ± 0.02 μm/s) (Figure 5C). The pausing time of BDNF vesicles was also significantly shorter in *Hdh*^Q111/Q111 ^neurons in which calcineurin was silenced (Figure 5D). The scramble RNA had no effect on vesicular velocity in wild-type and mutant condition, as shown by comparison with DMSO treatment (compare Figure 3, Figure 5 and Supplemental Figure 1). Finally, the dynamic parameters of transport in *Hdh*^Q111/Q111 ^neurons silenced for calcineurin were found to be similar to those in neurons from *Hdh*^+/+ ^mice treated with scramble RNA (anterograde velocity: 0.32 ± 0.03 μm/s and retrograde velocity: 0.34 ± 0.04 μm/s), consistent with the full rescue of transport to wild-type levels. Our findings demonstrate that calcineurin inhibition, whether by genetic methods or pharmacological methods based on FK506, restores the transport of BDNF vesicles, which is defective in HD, and therefore, is of therapeutic interest.

### HD mice display high levels of calcineurin activity

**Figure 6:**
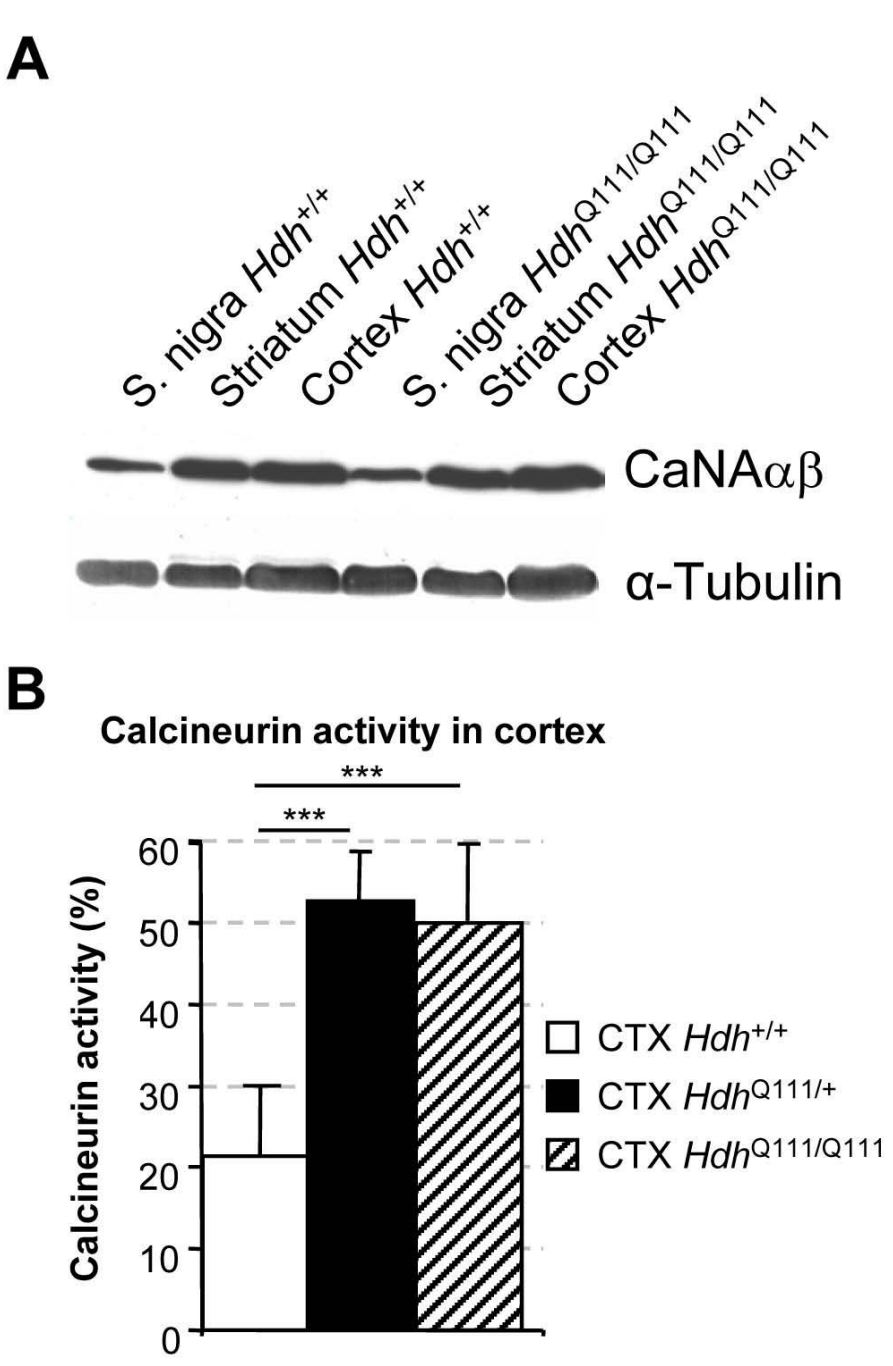
**Calcineurin activity but not protein levels are increased in *Hdh*^Q111/Q111 ^mouse brains**. A) Calcineurin levels were analyzed in cortex, striatum and substantia nigra from *Hdh*^Q111/Q111 ^and *Hdh*^+/+ ^mouse brains. No differences in protein levels were detected between wild type and HD condition. Note that calcineurin levels are lower in substantia nigra compared to cortex and striatum. (B) Calcineurin activity is significantly increased in the cortex of *Hdh*^Q111/Q111 ^and *Hdh*^Q111/+ ^mice compared to *Hdh*^+/+ ^mice. Data are from 6 mice per condition and were expressed as percentage of calcineurin activity per gram of tissue. (***p < 0.001 for *Hdh*^Q111/+ ^and *Hdh*^Q111/Q111^). No differences were found between *Hdh*^Q111/+ ^and *Hdh*^Q111/Q111 ^(p = 0.99; NS).

We previously reported that huntingtin is phosphorylated by Akt and that Akt is altered in *post mortem *brain samples from HD patients [14] and during disease progression [18]. This suggested that defects in Akt signaling pathway might be involved in the low level of huntingtin phosphorylation observed in HD [15,17]. In addition, this low level of phosphorylation may also be due to the dysregulation of calcineurin in HD [15]. Calcineurin is very abundant in brain, accounting for about 1% all the proteins present in this organ [34]. We investigated calcineurin levels in HD, by analyzing three regions of the brain -- the striatum, cortex and subtantia nigra -- based on the relevance of these regions to striatal afferences, BDNF transport and degeneration. The striatum is the region most affected in HD, and previous studies in cells of striatal origin have reported calcineurin dysregulation [19]. The cortex is the major source of BDNF for the striatum, via the cortico-striatal projections [35,36] and these neurons also degenerate in HD [37]. We also analyzed the substantia nigra, as neurons from this region project onto the striatum via the nigrostriatal pathway, which is also defective in HD, although this region is only a minor supplier of BDNF to striatal neurons [7,38,39]. Dissections were carried out on one-year old mice, as *Hdh*^Q111/Q111 ^mice manifest neurological symptoms at this time point [40]. Surprisingly, western blot analyses showed no difference between *Hdh*^+/+ ^and *Hdh*^Q111/Q111 ^mice in terms of the levels of calcineurin A catalytic subunit in these three regions (Figure 6A). As the cortex contains large amounts of calcineurin and is the major supplier of BDNF to the striatum, we determined the calcineurin activity in the cortex, as described above (Figure 4). We found that calcineurin accounted for 21 ± 8.31% of total phosphatase activity in *Hdh*^+/+ ^mouse cortex (Figure 6B). Surprisingly, levels of calcineurin activity were found to be significantly higher in *Hdh*^Q111/Q111 ^and *Hdh*^Q111/+ ^mice, at 49.85 ± 10.14% (Figure 6B). Thus, higher levels of calcineurin activity are observed in the brain during HD, suggesting that calcineurin dysregulation may contribute to HD pathogenesis by decreasing huntingtin phosphorylation at S421.

## Discussion

We demonstrate here that calcineurin is dysregulated in Huntington's disease and that the pharmacological and genetic inactivation of calcineurin leads to an increase in the phosphorylation of mutant huntingtin at S421, resulting in the restoration of its function in the intracellular transport of BDNF within cortical neurons.

Our results provide the first demonstration of a direct role of calcineurin in the regulation of vesicular transport. Huntingtin regulates the MT-dependent transport of organelles in neurons [3,10,12,33] and this function is regulated by phosphorylation in both physiological and pathological conditions [12,13]. We found that mutations mimicking huntingtin phosphorylation restored the ability of mutated huntingtin to transport BDNF. Also, we found that promoting huntingtin phosphorylation by activating the IGF-1/Akt pathway, leading to huntingtin phosphorylation at S421, restored the function of the mutant huntingtin protein in MT-dependent transport [13]. Our results demonstrating that calcineurin inhibition increases S421 phosphorylation and transport also provide support for the notion that huntingtin phosphorylation at S421 is critical for regulation of the function of this protein in transport and for neuronal death in HD [12,13]. Consistent with these data, FK506 exerts neuroprotective effects in neurons expressing polyQ-huntingtin [15]. Our observation that calcineurin inhibition restores BDNF trafficking and supply in the striatum in HD is consistent with previous studies showing that calcineurin inhibitors, such as cyclosporin A and FK506, decrease the level of neuronal cell death in the hippocampus after forebrain ischemia in animal models [41,42]. BDNF was reported to mediate the neuroprotective effect of calcineurin inhibitors, as shown by the selective induction of BDNF in the hippocampus of cyclosporine treated animals [43]. The induction of BDNF was linked to the increase in pCREB levels and BDNF transcription, but our results suggest that calcineurin inhibition could also increase BDNF trophic support by stimulating the transport of vesicles containing BDNF. Our results indicate that further studies of the general role of calcineurin in the control of intracellular trafficking in health and disease are warranted.

Our results validate calcineurin as a target for treatment in HD. We have demonstrated a specific change in calcineurin activity in disease conditions. Indeed, although calcineurin protein levels are similar in mouse brains containing the wild-type and mutant HD, significantly higher levels of calcineurin activity were observed in the cortex of mutant mice. This dysregulation is consistent with the downregulation of RCAN1-1L, a negative regulator of calcineurin in brains affected by HD [20] and the disturbance of calcium concentrations in HD [44]. In addition, activation of calcineurin could be induced by the cleavage of its specific inhibitor cain/cabin1 following the activation of the protease calpain [45]. Indeed, calpain activity is increased in HD brains and contributes to HD pathology [46,47]. Further evidence for the specific dysregulation of calcineurin in HD is provided by our observation that the treatment of neurons expressing wild-type huntingtin with FK506 had no effect on vesicular transport, whereas this treatment had a strong effect in neurons expressing the mutant protein. We have also shown that calcineurin activity is strongly inhibited by FK506 in neurons from HD mice. Thus, drugs aiming to block calcineurin activity are likely to be effective at treating the disease. Finally, the inhibition of calcineurin through two approaches, pharmacological and genetic in nature, resulted in similar beneficial effects, with an increase in the levels of phosphorylated huntingtin and correction of the axonal transport defect observed in HD.

## Conclusion

This study sheds light on the molecular mechanism by which calcineurin inhibition blocks neuronal death in HD. Our results extend the therapeutic potential of calcineurin inhibitors such as FK506, an FDA-approved drug capable of crossing the blood-brain barrier, and provide evidence in favour of clinical trials of the use of such compounds in HD patients.

## Methods

### Statistical analyses

Statview 4.5 software (SAS Institute Inc., Cary, NC) was used for statistical analysis. Data are expressed as mean +/- S.E.M.

### Constructs and siRNA

The plasmid encoding BDNF-mCherry was a generous gift from G. Banker (Oregon Health and Science University, Portland, Oregon). BDNF-mCherry shows cellular localization, processing, and secretion properties indistinguishable from those of endogenous BDNF. The wild-type and polyQ huntingtin constructs 480-17Q, 480-68Q, have been previously described [48] and correspond to human-mouse hybrids derived from mouse huntingtin cDNA: first exon of mouse huntingtin has been substituted by the homologous human one in mouse full length cDNA [49]. The siRNAs targeting mouse huntingtin correspond to the coding region 361-380 (siRNA1) of huntingtin mouse mRNA (GenBank Acc. n° XM_132009). The siRNA sequences targeting rat CaNAα and CaNAβ correspond to the coding regions 677-695 (GenBank Acc. No. NM 017041) and 448-466 (GenBank Acc. No. NM 017042) respectively. The scramble RNA (scRNA) control (Eurogentec, Seraing, Belgium) used has a unique sequence which does not match to any sequence in the genome of interest.

### Animals

Except for the experiments assessing BDNF transport in rat cortical neurons, all the videomicroscopy experiments and the biochemical analyses were conducted using *Hdh*^Q111/+ ^*Hdh*^Q111/Q111 ^and the corresponding *Hdh*^+/+ ^mice in the CD1 background. *Hdh*^Q111 ^knock-in mice, a generous gift from M.E. MacDonald, have been previously described [50]. For neuronal cultures from rat embryos, time pregnant Sprague-Dawley rats were obtained from Charles River Laboratories (Les Oncins, France). For experiments requiring brain dissection (Western blotting analyses and calcineurin assays), mice were deeply anaesthetized in a CO_2 _chamber, and their cortices, substantia nigra and striata were dissected out on ice and rapidly frozen using CO_2 _pellets. All experimental procedures were performed in strict accordance with the recommendations of the European Community (86/609/EEC) and the French National Committee (87/848) for care and use of laboratory animals.

### Neuronal cultures and transfection

Primary cortical neurons from E17 rat or from *Hdh*^Q111/Q111 ^or *Hdh*^+/+ ^mouse E15 embryos were prepared, cultured in Neurobasal B27 and transfected as described [3,14,48]. Cortical neurons were electroporated with the rat neuron Nucleofector^® ^kit according to the supplier's manual (Amaxa, Biosystem, Köln, Germany). For huntingtin gene replacement strategy, neurons were co-electroporated with siRNA1 and the 480-17Q or 480-68Q plasmids. For FK506 treatment, cells were treated with FK506 (0.1, 0.3, 1 μM; Alexis, Lausen, Switzerland) or vehicle (DMSO) for 30 min before videomicroscopy. All the experiments and in particular the videoexperiments were conducted in conditions in which no overt toxicity of the various constructs nor aggregation could be detected. The co-expression of BDNF and the various constructs as well as RNAi efficiency was verified by immunostaining after the videomicroscopy experiments. 95% or more of co-expression was observed. Single cell analysis of immunostaining levels revealed no difference in huntingtin expression between the different constructs nor signs of apoptosis in the conditions used.

### Videomicroscopy experiments and imaging treatment

Videomicroscopy experiments were done 2-3 days after transfection. Cells were cotransfected with BDNF-mCherry and various constructs of huntingtin or the corresponding empty vectors with a DNA ratio of 1:4. Live videomicroscopy was carried out using a Leica DM IRBE microscope and a PL APO oil 100× objective with a numerical aperture of 1.40-0.70, coupled to a piezo device (PI) and recorded with Photometrics CoolSNAP HQ2 camera (Roper Scientific, Trenton, NJ) controlled by Metamorph software (Molecular Devices, Sunnyvale, CA). Stacks were acquired in cultured medium at 37°C for cortical neurons. Images were collected in stream set at 2 × 2 binning with an exposure time of 100 ms (frequency of 2s) with a Z-step of 300 nm. All stacks were treated by automatic batch deconvolution using the PSF of the optical system, Meinel algorithm with parameters set at 7 iterations, 0.7 sigma and 4 frequency. Maximal z and time projection, animations and analyses of vesicles tracking were done with ImageJ software as previously described [33]. Supplemental movies and kymographs were obtained by collecting images at a frequency of 1 image/s with an acquisition time of 300 ms. The kymostacks were generated using a homemade ImageJ KymoToolbox plugin , NIH, USA; available on request at Fabrice.Cordelieres@curie.fr). For image analyses of fixed samples, images were acquired at room temperature with a Leica DM RXA microscope with a PL APO oil 100× NA of 1.4 objective coupled to a piezo device (PI) and a Micromax RTE/CCD-1300-Y/HS camera controlled by Metamorph software. The mounting medium was 0.1 g/ml Mowiol 4-88 (Calbiochem, Merck Biosciences, Darmstadt, Germany) in 20% glycerol. Z-stack was of 200 nm. Deconvolution was performed as for videomicroscopy.

### Western blot analysis

For neuronal cultures, neurons were washed with ice-cold PBS before scraping and lysis. In the case of experiments assessing the efficient silencing of huntingtin or calcineurin, half of the coverslips were lysed after videomicroscopy. For analyses of calcineurin in different brain regions, dissected frozen samples were directly resuspended in lysis buffer. Composition of buffers and all the procedures were performed as previously described [15]. 6% and 10% acrylamide gels were respectively used for huntingtin and calcineurin detection. 8% acrylamide gels were used to detect the two proteins on the same blot. Membranes were blocked in 5%BSA/TBST buffer (20 mM Tris-HCl, 0.15 M NaCl, 0.1% Tween 20) and immunoblotted with anti-CaN Pan A (1:1000; Chemicon) or anti-α-tubulin (1:10000; DM1A; Sigma, St Louis, MO), p150^Glued ^(clone 1, BD Biosciences, San Jose, CA, USA), home made anti-phospho-*htt*-S421-714 [15] and anti-huntingtin antibody mAb 4C8 (1:5000; clone 1HU-4C8, [49]) antibodies for 1 h. Membranes were then labelled with secondary IgG/HRP antibodies (Jackson ImmunoResearch, WestGrove, PA, USA), washed and incubated for 2 min with SuperSignalWest Pico Chemiluminescent Substrate (Pierce, Erembodegem, Belgium) according to the instructions of the supplier. Membranes were exposed to Kodak (Rochester, NY) BioMax films and then developed. Quantification of the signal was performed by densitometric scanning of the film using GelPRO analyzer software.

### Immunofluorescence

To ensure efficient silencing in electroporated neurons used for videomicroscopy, coverslips were fixed after videorecording with methanol for 3 min, blocked 1 h in PBS 1% BSA and incubated with mAb 4C8 anti-huntingtin antibody (1:100; [49]) during 90 min. Coverslips were rinsed three times in PBS 1:1000 tween 20 and incubated with a secondary Alexa 488 fluorescent antibody (Invitrogen, Oregon, USA). After incubation with DAPI (1:10000 in PBS; Roche, Indianapolis, USA), coverslips were rinsed three times and mounted with Mowiol.

### Calcineurin activity

Calcineurin activity was measured in primary cultures of cortical neurons from *Hdh*^Q111/Q111 ^mice, and in samples obtained from the cortex of 1 year old wild-type *Hdh*^+/+ ^and mutant *Hdh*^Q111/Q111 ^and *Hdh*^Q111/+ ^mice using the Calcineurin Cellular Activity Assay Kit (Calbiochem, San Diego, CA, USA). For experiments on cultures, neurons were treated with FK506 or DMSO and then lyzed in the buffer supplied by the manufacturer. For cortex analyzes, samples obtained from brain dissection were homogeneized in the supplied buffer. For both experiments, samples were processed according to the protocol provided by the manufacturer. Calcineurin activity was determined as the difference between total phosphatase activities minus the phosphatase activity in presence of 10 mM EGTA that blocks calcineurin activity. Data were expressed as the percentage of the total phosphatase activity.

## Abbreviations

The abbreviations used are DIV: days *in vitro*; BDNF: brain-derived neurotrophic factor; HD: Huntington's disease; MT: microtubule; O.D.: optical density; polyQ: polyglutamine; S421: serine 421.

## Competing interests

The authors declare that they have no competing interests.

## Authors' contributions

JRP, RP, SH and FS designed the experiments. JRP, RP, DZ and HY performed the experiments. JRP, RP, DZ, SH and FS analyzed the data. JRP, RP, SH, and FS wrote the paper. All authors read and approved the final manuscript.

## Supplementary Material

Additional file 1 **FK506 increases BDNF vesicular transport in rat cortical neurons expressing polyQ-huntingtin.** Representative movies showing dynamics of BDNF-mCherry vesicles in neurites from rat cortical neurons ectopically expressing the first 480 amino acids of huntingtin containing 68Q repeats (480-68Q, mutant). The upper movies shows DMSO treated neurons and the lower movies neurons treated with 1 µM FK506 during 30 min. For each movies, 6 randomly chosen vesicles were tracked and visualized as dots using ImageJ software. Videomicroscopy experiments were done as in Methods except that images were collected during 2 minutes at a frequency of 1 image/s with an acquisition time of 300 ms. Scale bar corresponds to 5 µm. Click here for file

Additional file 2 **FK506 treatment increases transport of BDNF-containing vesicles in cortical neurons from HdhQ111/Q111 mice.** Representative movies showing the effect of FK506 treatment on the dynamics of BDNF-mCherry vesicles in neurites from cortical primary neurons from knock-in HdhQ111/Q111 mice. The upper movies shows DMSO treated neurons and the lower movies neurons treated with 1 µM FK506 during 30 min. For each movies, 6 randomly chosen vesicles were tracked and visualized as dots using ImageJ software. Videomicroscopy experiments were done as in Methods except that images were collected during 2 minutes at a frequency of 1 image/s with an acquisition time of 300 ms. Scale bar corresponds to 5 µm. Click here for file

Additional file 3 **FK506 does not modify the velocity of BDNF-containing vesicles in cortical *Hdh*^+/+ ^mice neurons**. (A and B) Cortical primary neurons from wild type knock-in Huntington's disease mice model were processed as for *Hdh*^Q111/Q111 ^cells in Figure 3. Neurons were treated with either DMSO or the following increasing concentrations of FK506 0.1 μM, 0.3 μM 1 μM. No significant differences were found in both anterograde and retrograde velocities for all tested concentrations (Anterograde: p = 0.73; NS for 0.1 μM, p = 0.77; NS for 0.3 μM, p = 0.47; NS for 1 μM. Retrograde: p = 0.46; NS for 0.1 μM, p = 0.91; NS for 0.3 μM, p = 0.63; NS for 1 μM). Data are from two independent experiments, 3413 tracks, 13 cells for *Hdh*^+/+ ^+ DMSO, 3347 tracks, 13 cells for *Hdh*^+/+^+ FK506 0.1 μM, 3715 tracks, 12 cells for *Hdh*^+/+ ^+ FK506 0.3 μM, 2181 tracks, 8 cells for *Hdh*^+/+ ^+ FK506 1 μM. Click here for file
