# Supplementary figures and images for: Genetic and pharmacological inhibition of calcineurin corrects the BDNF transport defect in Huntington's disease
Source: Mol Brain. 2009 Oct 27;2:33. doi: 10.1186/1756-6606-2-33 (PMC2776580; doi:10.1186/1756-6606-2-33)

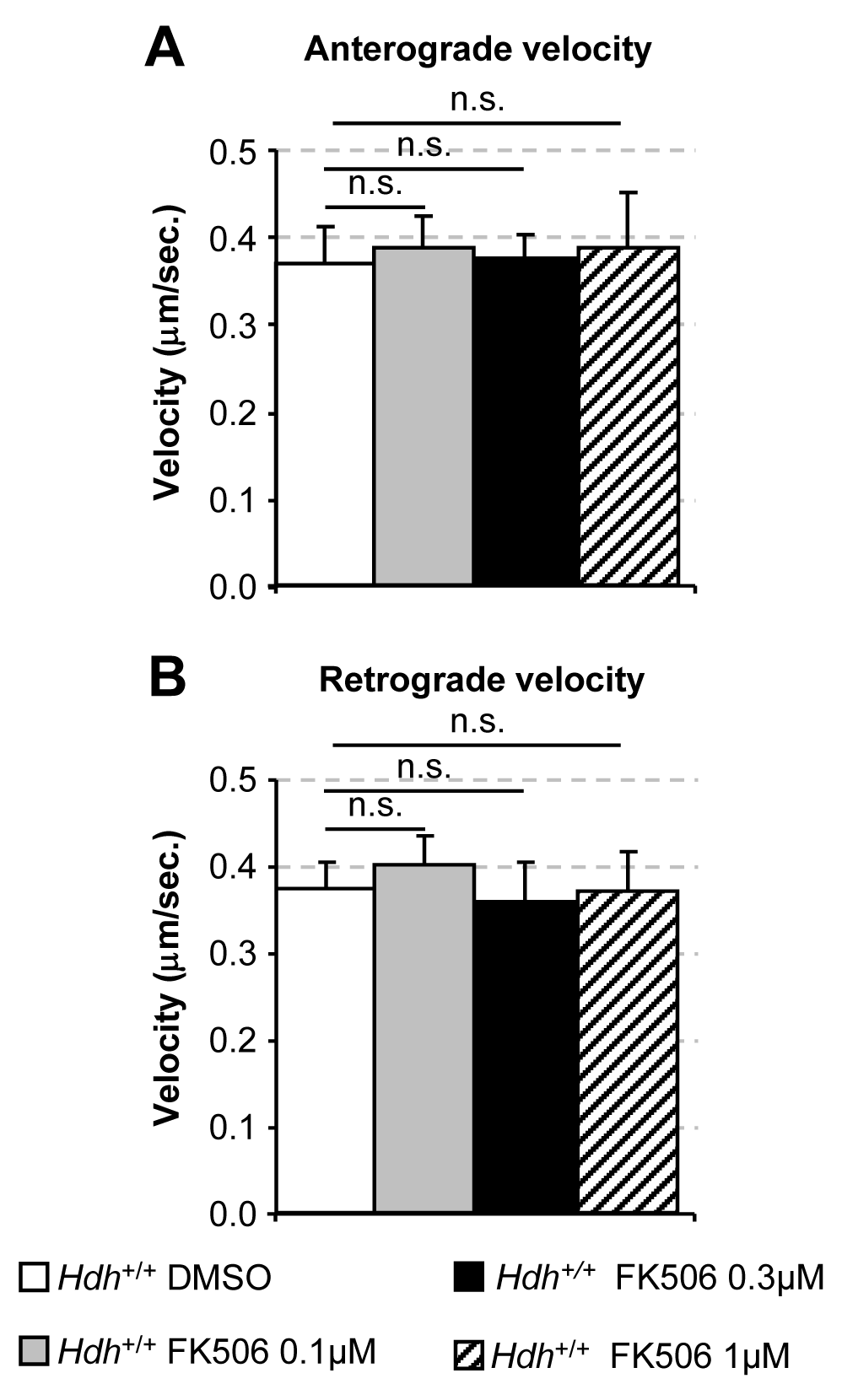

Supplement: Additional file 3 — FK506 does not modify the velocity of BDNF-containing vesicles in cortical Hdh+/+ mice neurons. (A and B) Cortical primary neurons from wild type knock-in Huntington's disease mice model were processed as for HdhQ111/Q111 cells in Figure 3. Neurons were treated with either DMSO or the following increasing concentrations of FK506 0.1 μM, 0.3 μM 1 μM. No significant differences were found in both anterograde and retrograde velocities for all tested concentrations (Anterograde: p = 0.73; NS for 0.1 μM, p = 0.77; NS for 0.3 μM, p = 0.47; NS for 1 μM. Retrograde: p = 0.46; NS for 0.1 μM, p = 0.91; NS for 0.3 μM, p = 0.63; NS for 1 μM). Data are from two independent experiments, 3413 tracks, 13 cells for Hdh+/+ + DMSO, 3347 tracks, 13 cells for Hdh+/++ FK506 0.1 μM, 3715 tracks, 12 cells for Hdh+/+ + FK506 0.3 μM, 2181 tracks, 8 cells for Hdh+/+ + FK506 1 μM. [file 1756-6606-2-33-S3.TIFF]
